# Supplementary material for: Splice-Junction-Based Mapping of Alternative Isoforms in the Human Proteome
Source: Cell Rep. Author manuscript; Available in PMC 2020 Jan 15. (PMC6961840; doi:10.1016/j.celrep.2019.11.026)
Supplement: 2 [file NIHMS1546469-supplement-2.zip › DF1/PXD010154/Testis/Testis_28_ART3_WSSCLGLLK.pdf]

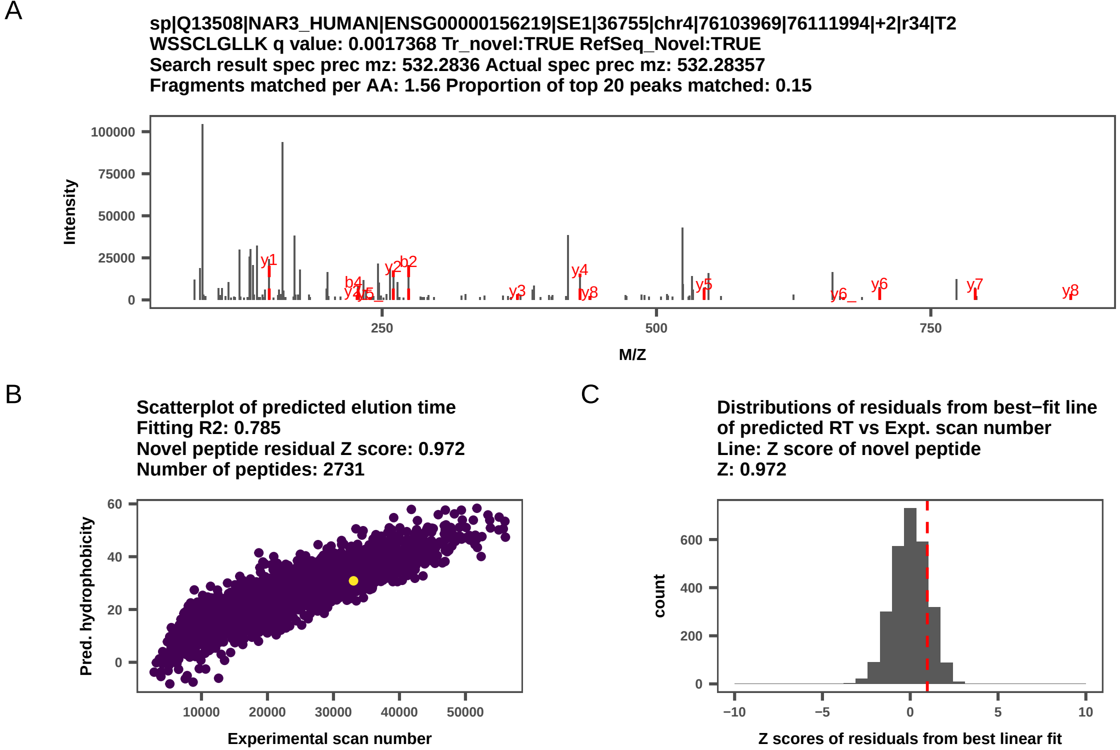
A
sp|Q13508|NAR3_HUMAN|ENSG00000156219|SE1|36755|chr4|76103969|76111994|+2|r34|T2
WSSCLGLLK q value: 0.0017368 Tr_novel:TRUE RefSeq_Novel:TRUE
Search result spec prec mz: 532.2836 Actual spec prec mz: 532.28357
Fragments matched per AA: 1.56 Proportion of top 20 peaks matched: 0.15
Intensity
100000
75000
50000
25000
0
y1
b4
y2
b2
y3
y4
y8
y5
y6_
y6
y7
y8
250
500
750
M/Z
B
Scatterplot of predicted elution time
Fitting R2: 0.785
Novel peptide residual Z score: 0.972
Number of peptides: 2731
Pred. hydrophobicity
60
40
20
0
10000
20000
30000
40000
50000
Experimental scan number
C
Distributions of residuals from best−fit line
of predicted RT vs Expt. scan number
Line: Z score of novel peptide
Z: 0.972
count
600
400
200
0
−10
−5
0
5
10
Z scores of residuals from best linear fit
